# Demand-only energetics at 120 ATP per glucose: A reply to Lynch

**DOI:** 10.1016/j.bbabio.2026.149587

**Published:** 2026-03-06

**Authors:** William F. Martin

**Affiliations:** Institute of Molecular Evolution, Heinrich Heine University Düsseldorf, 40225, Düsseldorf, Germany

**Keywords:** Energy in evolution, Mitochondria, Bioenergetics, Eukaryogenesis, Biosynthetic, costs Costs of a gene

## Abstract

Recently in these pages, a paper by Lynch appeared in response to a report showing that his numbers for biosynthetic costs (ATP demand) in cells are inflated, so much so that they would require *E. coli* to obtain >100 ATP per glucose and mitochondria to obtain >240 ATP per glucose. The inflated estimates trace to one factor: Lynch exclusively considers ATP demand and systematically neglects ATP supply—the essence of bioenergetics. Thermodynamics stipulate that a cell cannot grow if its ATP demands exceed its ATP supply. Here I compare Lynch's calculated ATP demands to laboratory measurements of the ATP supply that *E. coli* synthesizes during cell division. The results bear out my case, and leave no doubt: Lynch's calculations require *E. coli* to synthesize ~120 ATP per glucose, which is thermodynamically impossible. As a consequence, his demand-only ‘energetic’ attacks on mitochondria and endosymbiosis in evolution are baseless.

## 1 Introduction

In his recent response [1] to a report that his numbers for biosynthetic costs in cells are inflated [2], Lynch defends his theoretical calculations for ATP needed to synthesize an Escherichia coli cell as both correct and valid, even though they would demonstrably require *E. coli* to obtain >100 ATP per glucose, and mitochondria to obtain >240 ATP per glucose [2] during cell growth. Lynch presents a cost-only (demand-only) rendering of bioenergetics as ‘evidence’ that mitochondria were irrelevant to eukaryotic evolution [1,3–5] while disregarding ATP supply in cells [1], which is unconstrained [6] in his model [3,4]. Target of his attack [1] is the view that mitochondria were essential to eukaryotic origin [7–12]. The tenor and content of Lynch's response [1] require renewed inspection of his calculations, but this time using an approach independent of [2]. Clearly, a cell cannot grow if its ATP demands exceed its ATP supply—on that Lynch will have to agree. I therefore compare his theoretical ATP demands to laboratory measurements of the ATP supply that a growing *E. coli* cell actually synthesizes during cell division.

That the life process obeys the laws of thermodynamics, specifically Helmholtz's conservation of energy [13], has been known since Rubner's [14] 1902 measurements of the reactants and products plus heat produced during mammalian growth in calorimeters [15]. In the 1970s enough became known about the biosynthetic and ATP-producing pathways of *E. coli* for Stouthamer [16] to calculate the amount of ATP needed to synthesize an *E. coli* cell from glucose and mineral salts. Stouthamer's published ATP requirements for amino acids, nucleic acids, lipids and polysaccharides, including import, and using the standard value of 0.28 pg per *E. coli* cell, sum to 5.8 billion ATP per cell division (Table 3 of [2] and Table 5 of [16]).

Lynch calculates drastically higher ATP demands, for two reasons. First, he disregards ATP synthesis in cells [1,3–5], assuming that ATP is somehow available in real cells for free [6], which is never true. Second, he bases his calulations not on standard biosynthetic costs in terms of how many net ATP-consuming steps are involved in biosynthesis from glucose in the tradition of Stouthamer [16] and Palsson [17], but on a theoretical calculation [18] and an inflationary method of cost estimation, the Craig and Weber (CW) method [19]. As explained previously [2], the CW method [19] calculates biosynthetic cost as the amount of ATP needed to synthesize a compound from universal intermediates like pyruvate or erythrose-4-phosphate plus the amount of ATP that the cell could have gained had it not synthesized the compound [19], respiring the biosynthetic intermediates instead [2]. One cannot overemphasize the point: By not synthesizing amino acids, bases and cofactors from glucose, respiring the glucose instead, *E. coli* can obtain ATP, but it cannot grow for lack of biosythetic building blocks [2]. Therefore none of the organisms for which Lynch has calculated ‘costs’ using the CW method can grow. Yet all of Lynch's ‘energetic’ calculations are for maximum growth rates [3,4]. There might be one or the other newly arisen regulatory mutant *E. coli* cell in an exponentially growing culture [17,20] that respires all of its glucose rather than making amino acids and bases, but such cells cannot grow, for lack of amino acids, hence they do not contribute to biomass and are therefore irrelevant, also in evolution. Cells are >50% protein [16], they require amino acids for growth. Lynch is exclusively interested in costs (demand), but by over-looking supply, his calculations are unconstrained.

## 2 Getting the facts straight

**Fig. 1:**
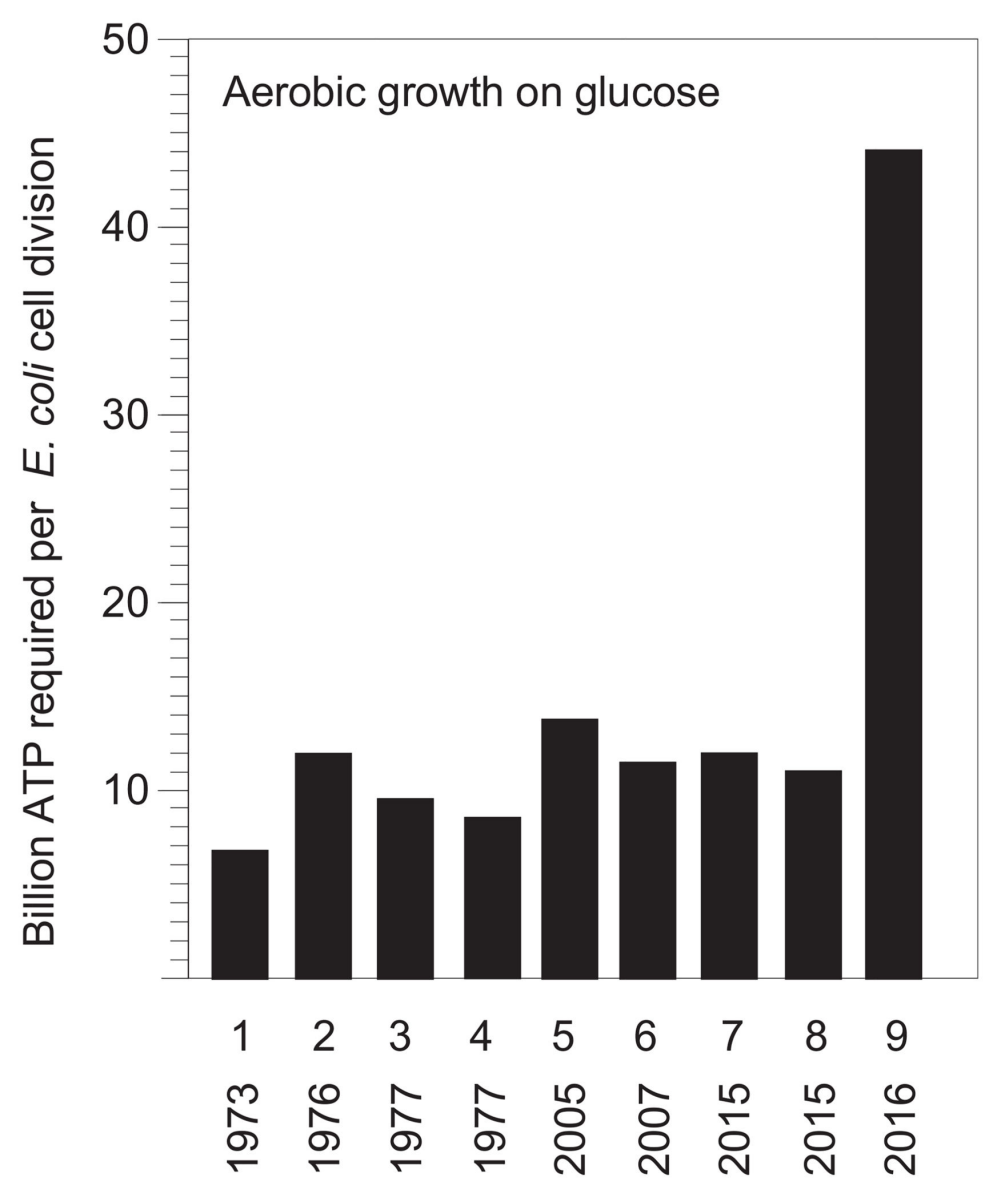
Supply (bars 1–8) vs demand (bar 9) for aerobic *E. coli* growth on glucose. ATP supply is measured as Y_ATP_ and plotted on the Y axis as billion ATP per cell division, which includes biosynthetic costs (ca. 88%) and maintenence energy (ca. 12%) [17]. For literature values expressed as mmol ATP per gram dry weight, multiply by (6 ⋅10^23^ ATP/mol)⋅(1 mol/10^3^ mmol)⋅(2.8 × 10^–13^ g/cell); 60 mmol ATP per g converts to 10 billion ATP per cell. For literature values expressed as grams dry weight per mol ATP, invert and multiply by 6⋅10^23^ ATP/mol)⋅(2.8 × 10^–13^ g/cell); 16.7 g per mol ATP converts to 10 billion ATP per cell. Year of publication is shown under the column. **1** [16] Stouthamer's ATP demand +12% maintenance energy, for comparison; **2** [35], 13.9 g/mol ATP; **3** [36] 17.6 g/molATP, glucose limited; [36], 20.0 g/molATP, ammonium limited; **5** [37] 11.6 g/mol ATP, glucose limited; **6** [17], 68.2 mmol ATP/g; **7** [32] 10 billion ATP per division +12% maintenance energy. **8** Fig. 3 of ref. [38], 15 g/mol ATP. **9** [3,4] biosynthetic ATP demand calculated using the CW method, see text and Table 1, + 12% maintenance energy.

Lynch states that he does not understand how I came to the conclusion that his cost estimates are inflated ([1], p. 149576 left column top). To explain, in physiology, ATP supply needs to meet or exceed ATP demand. Furthermore, biosynthetic numbers need to be derived, not declared. In his response [1], Lynch declares that the synthesis of an amino acid costs 6 ATP, without source or explanation ([1] p. 149576 left column, top). The number is furthermore incorrect, as shown in Table 4 of reference [16] and in Table 1 and Fig. 1 of reference [2], because *E. coli* synthesizes net ATP from glucose during amino acid synthesis [2,16] such that the ATP demand (cost) of synthesizing an average amino acid in *E. coli* from glucose is 0.28 ATP per amino acid [2,16]. Lynch, using the CW method, neglects ATP synthesis from glucose and writes that the total cost of an amino acid is actually “*30 ATP*”, because an “*embellishment*” can be calculated [1]. Lynch requests a reasoned discussion [1], so we turn to the causes of problems with his calculations [1,3–5,21,28–30].

To start, Stouthamer [16] does not report an energy requirement for *E. coli* of 20–60 billion ATP per cell, as the abstract of the paper at the root [18] of all of Lynch's calculations [3,4] asserts. Instead, Stouthamer [16] reports in his Table 5 (first column, bottom number) that the synthesis of one gram of *E. coli* cells grown on glucose and inorganic salts, including ATP expense for transport requires 347⋅10^–4^ mol ATP per g of cells (dry weight). To convert that value to ATP per cell, we multiply by 6.02⋅10^23^ molecules of ATP per mol ATP and by the mass of an *E. coli* cell, typically 2.8⋅10^–13^ g per cell, on which we agree [3], which yields 5849⋅10^6^ ATP per cell, or 5.8 billion ATP per cell (including transport). A comparison of the values that Stouthamer, an expert for *E. coli* metabolism [16], and Lynch, an expert for population size [22], obtain for biosynthetic costs is given in Table 1.

What is the source of the inflation in estimates for biosynthetic costs in Table 1? Lynch adds non-existent ATP costs to the synthesis of monomers by using the CW method: the cost of synthesis, which is real, plus the potential yield from respiration [19], which is not [2]. During *E. coli* exponential growth on glucose, the amount of glucose that can be potentially redirected to respiration and the amount directed to amino acid synthesis are not variables that can be arbitrarily manipulated [1]. They are hard-wired into wild-type metabolism, hence the growth process [17]. To illustrate, if *E. coli* were to respire all of its glucose to make ATP, it would have no carbon for synthesis of amino acids, bases, lipids or saccharides, therefore it would be unable to grow. Similarly, if *E. coli* were to devote all of its glucose to amino acid, base, lipid and saccharide synthesis, it would immediately exhaust its ATP supply and again be unable to grow. Glucose respiration (ATP synthesis) and cell biosynthesis (ATP hydrolysis) have to balance in an overall stoichiometry that supports continuous growth [17]. If wildtype *E. coli* has decided to grow, it uses regulated gene expression and allosteric regulation of enzymes to direct the available carbon source into routes that balance biosynthetic demand with bioenergetic ATP supply, while leaving enough ATP to cover non-biosynthetic ATP demand (maintenance energy) [17,24]. Such finely tuned metabolic regulation coordinates hundreds of enzymatic activities into a unified output—growth [25]—and is the product of natural selection, not population size [22], operating on regulatory genes [25] and regulated enzymes [26] during evolution. In this respect, what is true for *E. coli* is true for the elephant.

The result is metabolism, a set of reproducible enzyme activites that yield growth from the regulated and balanced allocation of resources. Any short-term deviations in those relative allocations are corrected by regulation—the guardrail that maintains the cell in redox balance. Redox balance is essential [27]: the number of electrons that enter the cell has to equal the number that leave the cell, otherwise metabolism comes to an immediate halt, for example during suffocation. Although the true costs for the synthesis of an average amino acid in *E. coli* are 0.28 ATP [16] (Table 1), Lynch treats the cost of 23.55 or 30 ATP per amino acid as true ATP demand and surmizes [1] that “*By dividing such costs by the total lifetime cost of building / maintaining the cell, one arrives at a fractional reduction in the cell-division time associated with the embellishment*.” Again, Lynch simply overlooks supply. Cell division time is not a function of cost (ATP demand), it is a function of supply, because during growth the ATP supply can be a rate-limiting variable, while ATP demand is a constant, given substrates. For example, anaerobic growth of *E. coli* generally involves longer doubling times because of energetic efficiency and ATP synthesis rates in mixed acid fermentation; the ATP demand is the same with and without O_2_ because under aerobic and anaerobic conditions *E. coli* uses the same biosynthetic enzymes (demand), but different energy-conserving ATP synthetic routes (supply) [24].

## 3 Getting the facts straight on *E. coli* biosynthetic costs (demand)

We start with protein. Stouthamer's [16] Table 5 rightly reports the synthesis of amino acids in one gram of *E. coli* cells from glucose and ammonium as requiring 13.55⋅10^–4^ mol ATP per g (= 0.23 billion ATP per cell). This converts to 0.28 ATP per amino acid on average. This value is <1 because *E. coli* obtains net ATP from the synthesis of several amino acids from glucose [2,16]. The cost of amino acid polymerization is 4 ATP per peptide bond or 191⋅10^–4^ mol ATP per g dry weight (= 3.2 billion ATP per cell) or 55% of the total ATP expense. In all papers recently criticized [2], Lynch and colleagues [28–30] erroneously take the cost of amino acid synthesis as 23.55 ATP per average amino acid—84 times higher than the true biosynthetic cost from glucose in *E. coli*, 0.28 ATP per amino acid on average. If the reader is in doubt, the 23.55 number can be found in Supplementary Table 3 of [4], although Lynch currently uses 30 ATP per amino acid [1,21]. At the lower 23.5 ATP per amino acid biosynthetic cost, protein synthesis in *E. coli* would require 3.2 billion ATP per cell (peptide bonds) plus 19.32 billion ATP per cell according to Lynch, for a total of 22.5 billion ATP per cell only for protein (Table 1).

Nucleic acids also exhibit inflated costs in Lynch's ‘energetic’ accounting. Stouthamer (Table 4 of ref. [16]), calculates that an average base in RNA costs 7.5 ATP for synthesis and 2 ATP for polymerization (0.38 billion ATP plus 0.153 billion ATP per cell). In DNA the costs are 9 ATP per base plus 2 per polymerization (0.144 billion plus 0.032 billion ATP per cell). The numbers are still valid. For mRNA turnover no new bases are synthesized, only polymerization cost are incurred (0.23 billion ATP), yielding 0.94 billion ATP per cell for nucleic acids. Lynch calculates nucleotide biosynthesis at the flat rate of 50 ATP per base ([3], page 165691). With polymerization and turnover constant, this increases Lynch's nucleic acid synthesis cost above the real value to 2.69 billion ATP for RNA, 0.83 billion for DNA and 0.23 billion ATP for mRNA turnover for a total of 3.75 billion ATP per cell (Table 1).

For the synthesis of lipids from glucose, Stouthamer [16] (p. 551) points out that ATP is gained during glycolysis to pyruvate such that “*the formation of 1 mole of phosphatidyl ethanolamine with two C16 fatty acids requires only 1 mole of ATP per mole*” yielding lipid costs of 0.023 billion ATP per cell. The CW method overlooks ATP that *E. coli* gains along pathways from glucose to biosynthetic intermediates like pyruvate. For lipids, Lynch [4] estimates 240–640 ATP per lipid molecule across lineages while highlighting the (in his view) exorbitant costs of lipids. Using the value of 367 ATP per lipid molecule for *E. coli* (ref. [4], Appendix 1, Table 4) and 1.4⋅10^–4^ mol lipid monomers per gram of cells [16], we obtain 23.5⋅10^–6^ lipid molecules per cell, times 367 ATP per lipid molecule equals 8.6 billion ATP per cell for lipids according to Lynch (Table 1).

For the synthesis of polysaccharides (cell wall and glycogen, 16.6% of dry weight), Stouthamer [16] notes a biosynthetic cost of 2 ATP per monomer, or 0.34 billion ATP per cell. Lynch provides no values for glycogen or polysaccharide content [3,4], 16% of the mass of a typical exponentially growing *E. coli* cell. Nowhere does the inapplicability of the CW method become more evident than with glycogen. Using the CW method, the cost of synthesizing glycogen is 2 ATP per monomer plus maximally 29.6 ATP per glucose (26.6 ATP per glucose from chemiosmotic ATP synthesis [2] plus four from substrate level phosphorylation, minus one for import), yielding a cost in Lynch's CW accounting of ~30 ATP per monomer, or 3.8 billion ATP for saccharides. Glycogen synthesis does not *cost* 30 ATP per glucose monomer polymerized, it yields 30 ATP per monomer when respired. The CW method is inapplicable to cell growth [2].

To the values for biosynthesis we have to add the cost of substrate import, which is 0.87 billion ATP per cell (Table 1) for direct comparison to Stouthamer's calculations. Summed up, Lynch's calculations thus deliver a total cost—an ATP demand—for synthesizing an *E. coli* from glucose, salts and O_2_ of 39.7 billion ATP per cell (Table 1), which is within the 20–60 billion range published using the CW method [18]. Lynch [1] states that Phillips and Milo [31] arrive at “*similar*” (inflated) estimates for the cost of biosynthesis in *E. coli* as he does, which is not correct. Phillips [32] (p. 199) reports a biosynthetic cost of 10 billion ATP per cell on glucose, not 40 billion. The paper by Milo and Phillips [31] to which Lynch refers, relies in turn on a website [https://openwetware.org/wiki/Ecoli_ATP_requirement, accessed Feb 2026 and saved], that reports 20 billion ATP synthesis (not demand) per division on glucose, but under anaerobic conditions; the energetic efficiency of anaerobes is on the order of 25–50%, whereas that of aerobic glucose respiration closer to 60% [33]. The same website states that it is “*unclear*” how the number of 20–60 billion ATP/cell for the biosynthesis of one *E. coli* cell was calculated; as shown here in Table 1, it was calculated using the CW method [2].

## 4 The test: supply vs. demand

Microbiologists and systems biologists have often measured the amount of cell mass, dry weight, that growing aerobic *E. coli* cultures produce from glucose under conditions where the substrate consumption can be accurately measured. This allows the amount of ATP produced for a given mass of *E. coli* cells to accrue during growth to be accurately estimated. The value is growth yield per ATP, or Y_ATP_. The units of Y_ATP_ are expressed as grams of dry cell mass per mol ATP, sometimes the reciprocal, mmol ATP per gram of dry cells is used, as in [16]. In Fig. 1, the units are converted to billion ATP per cell for convenience. Y_ATP_ is a measured value from laboratory experiments and based on enzymatic knowledge about *E. coli* ATP synthetic routes. It takes into account both the ATP expended for biosynthesis plus the fraction of ATP consumption that is not directly associated with biosynthetic pathways, a quantity called maintenance energy, which is not easy to measure directly. Modern studies estimate that maintenance energy comprises about 12.3% of *E. coli’s* total ATP budget under continuous aerobic growth on glucose [17]. That estimate for maintenance energy, rounded here as 12% of the total ATP budget, leaves 88% for biosynthesis. To compare Lynch's calculations for the cost of growth (ATP demand) to measured growth yields (ATP supply), we have to add 5.3 billion ATP for maintenance (12% of the total ATP expense) to his biosynthetic cost of 38.7 billion ATP per cell, yielding 44 billion ATP per cell required for growth, including maintenance (Fig. 1).

Can *E. coli* ATP supply meet that demand of 44 billion ATP per cell? No. Typical measured values of Y_ATP_ for aerobic growth of *E. coli* on glucose are on the order of 16 g/mol (16 g of cells dry weight per mol of ATP synthesized), which converts to 10.5 billion ATP per cell (Fig. 1). Those numbers represent the supply of ATP available to the real, growing cell for biosynthesis and growth. They exceed the original estimate of 5.8 billion ATP per cell from Stouthamer (6.6 billion ATP per cell including maintenance) [2,16], but they fall far short of the 44 billion ATP per cell demand of Lynch (Fig. 1), exceeding *E. coli*'s ATP supply by a factor of 4.

## 5 This has consequences

Life is a chemical reaction. Under a demand of 44 billion ATP per division, an *E. coli* cell with a dry weight of 0.28 pg grown in a culture with measured glucose consumption, measured O_2_ consumption, and measured cell mass accumulation cannot alter its size, density, or composition, because these are known physical values [32]. Growing at a demand of 44 billion ATP per cell with a supply of 10 billion ATP per cell, *E. coli* would have no option but to synthesize 4 times more ATP per glucose than it really does, which is not possible, as pointed out previously [2], regardless of doubling time. Aerobic respiration releases –2872 kJ⋅mol^–1^ per mol of glucose [34], an amount of free energy from which efficient O_2_ respirers, and *E. coli* under optimal conditions, can glean roughly 30 ATP per glucose. A demand of four times more ATP per glucose would require *E. coli* to somehow extract 11,488 kJ per mol of C_6_H_12_O_6_ from glucose oxidation with O_2_, which is physicochemically not possible. On that all must agree.

This leaves no other conclusion than the obvious: Lynch needs to accept that his entire repertoire of demand-only ‘energetic’ calculations are incorrect, starting with reference [3] and snowballing forward. From that it follows that all of the ‘energetic’ arguments in his crusade against mitochondria [1,3–5,21,22,28–30] fail, because they are based on demand-only-no-supply energetics that require *E. coli* to obtain 120 ATP per glucose (Fig. 1), which is not possible, or mitochondria to obtain 240 ATP per glucose [2], which is also not possible.

Furthermore, all of Lynch's calculations are based on evolution and selection operating on organisms multiplying at their maximum growth rates [3,4]. No organism in nature evolves at its maximum growth rate. Microbiologists learn in college that an *E. coli* cell growing at its maximum growth rate (3 divisions per hour) will outweigh the Earth in less than 48 h because 0.28 pg per cell times 2^144^ cells represents more mass, let alone carbon, than the Earth has to offer. Evolution in the real world is not a “*shazam process*” [1] in which cells or organisms evolve at their maximum growth rate over geological time scales or synthesize 120 ATP per glucose.

Lynch justifies his inflated estimates by declaring that CW method and similar demand-only approaches are used in papers by “other scientists”, which is true, but with two important caveats. First, those papers also only consider ATP demand, neglecting ATP supply, and therefore fail to recognize that demand-only calculations [1,3,4,19] deliver ATP demands per glucose that do not match the ATP supply per glucose that growing organism can deliver (Fig. 1). Second, those papers were aiming to model the energy demands of cells, while Lynch is aiming to trivialize the significance of mitochondria [1,3,4].

## 6 And what about mitochondria?

Lynch's closing passages dismiss the idea that the symbiotic origin of mitochondria has any evolutionary significance [1]. This echos an anti-symbiotic sentiment that traces back to Wilson in 1928 [39]. Wilson condemned Wallin's 1925 [40] and Mereschkowsky's 1905 [41] ideas about the symbiotic origin of mitochondria and chloroplasts on one page of his famous 1200-page cell biology textbook [39]. Wilson's condemnation stuck for decades until Margulis [42] revived the idea in 1967, and even then, well into the 1970s prominent biologists lucidly explained in major journals how non-symbiotic mechanisms could readily derive mitochondria and chloroplasts (including their genomes) within a cell without the need for invoking the process of symbiosis in evolution at all [43–46]. That 1970s sentiment is echoed in Lynch's reference to “*other models*” of eukaryogenesis that do not involve mitochondria [1].

As a case in point, Lynch offers the prediction that *E. coli* grown in a continuous culture bioreactor should have “*excess energy*” and thus reveal a “*substantial increase in cellular complexity*” after “*a few years*” [1]. The statement is untrue. First, Lynch mistakenly states that growing *E. coli* cells have “*excess energy*,” they do not have excess energy, they have ~10 billion ATP per cell division (Fig. 1) and continuous growth. Second, no one ever reported *E. coli* turning into yeast in 75,000 generations of continuous culture observations over decades [20]. Third, Lynch's gradualist *Gedankenexperiment* has already been done in nature. How so? It has been estimated that roughly 10^40^ prokaryotic cells have ever existed on Earth [12], others arrive at the same number [47]. Thus, 10^40^ prokaryotic cells have had 4 billion years of opportunity to become complex via Lynch's evolutionary mechanisms: point mutation, gene duplication, and population size effects [22]. It has been pointed out [12] that (i) only one of those 10^40^ prokaryotic cells ever became the mitochondrion, that (ii) only one of those 10^40^ prokaryotic cells ever became its host cell, and that (iii) all eukaryotes on Earth indisputably descend from a cellular merger of those 2 out of 10^40^ prokaryotes [12], which is an observation [48,49], not a claim.

That is, I am saying that those two 1-out-of-10^40^ events are not only the same event, they are furthermore causally and mechanistically connected, for reasons of symbiosis (anaerobic syntrophy) bioenergetics and endosymbiotic gene transfer from symbiont to host [7–12]. Lynch is clearly saying that there is no connection whatsoever between mitochondrial origin and eukaryote origin [1,3–5] and that the coccurrence of mitochondria and cellular complexity in eukaryotes has no causal connection, it is pure coincidence [1]. Lynch's proposal makes the presence of mitochondria in eukaryotes the result of two spatiotemporally disjunct and mechanistically independent one-in-10^40^ events: A one-in-10^40^ origin of eukaryotes followed by an altogether unconnected one-in-10^40^ origin of mitochondria in some member of a preformed, primitively amitochondriate eukaryotic lineage [1], an idea popular in the 1980s (reviewed in [48,49]). Thus, Lynch has it that the presence of mitochondria in eukaryotes is the result of a one-in-10^80^ event, a pure chance coincidence of two independent one-in-10^40^ events during cell evolution; 10^80^ is roughly the number of protons in the universe. Lynch asks [1] whether my endosymbiotic model invokes “*special creation*”. Fifty years ago, endosymbiosis was indeed called a “*revival of special creation*” in theories that downplay the evolutionary significance of mitochondria [46], that outdated view resurfaces once more [1].

Lynch closes by saying “*Such a scenario would avoid the common but odd assumption that a hapless, previously free-living bacterial cell somehow became immediately enslaved by a governing host cell*.” To get the facts straight, in my theory, the host is not a dominant “*governor*” of a “*hapless*” mitochondrial slave, rather the symbiont is the biochemically stronger partner [7] because it imposes its carbon and energy metabolism upon a methanogenic host via mitochondrial energetics and via endosymbiotic gene transfer for pyruvate supply, the two become a eukaryote with bacterial lipids, with a facultatively anaerobic mitochondrion, with ATP-producing glycolysis in the cytosol and with a thermodynamically favourable heterotrophic lifestyle [7–12]. In estimating biosynthetic costs, we have to work with physiology and the facts, whereby thermodynamics constrain the possible—it’s the law.

## Figures and Tables

**Table 1:** Biosynthetic ATP demands (costs) of Stouthamer and Lynch per cell.

		ATP per monomer^a^				ATP per cell [billion]		
Constituent		[%]^b^	Stout.^c^	Lynch^d^		Stout.^c^	Lynch^d^	Inflation [−fold]^i^
Protein		52.4	0.28	23.5		3.42	22.5	84
RNA^e^		15.7	9.5	50		0.96	2.9	5.2
DNA		3.2	11	50		0.18	1.06	4.5
Lipid		9.4	1	367^f^		0.023	8.6	367
Polysaccharide		16.6	2	30^g^		0.34	3.8	15
Import		−	−	−		0.87	0.87^h^	−
Total requirement (billion ATP per cell):						5.8	39.7	

a Notes: Cost for synthesizing monomers from glucose and ammonia. Monomers are: Protein, amino acid; RNA and DNA, nucleoside monophosphate; lipids, phosphatidylethanolamine; polysaccharide, glucose.

b % by dry weight. Data from

c ref. [16],

d refs. [3, 4].

e including mRNA turnover.

d ref. [4], some of the numbers in [4] were taken from [23], with [4] subsequently versioned.

g Not reported by Lynch but calculated here using the CW method [19], see text.

h Not calculated by Lynch, Stouthamer's value of 5.208 mmol ATP per g of cells (Table 5 of [16]) is used.

i Fold inflation incurred by Lynch by using the CW method; inflation increases if the cell is an amino acid auxotroph, like mammals [2]. Stouthamer reported a requirement of ATP per gram, not per cell, so he did not assume a specific cell mass, 0.28 pg per cell is used here. Lipid synthesis is the most highly inflated cost, but protein synthesis is the major source of inflation [1,3,4] because cells are ~50% protein by weight [16].

## Data Availability

Data will be made available on request.

## References

[1] Lynch M (2026). Energetics and evolution: response to Martin. BBA-Bioenergetics.

[2] Martin WF (2025). ATP requirements for growth reveal the bioenergetic impact of mitochondrial symbiosis. BBA-Bioenergetics.

[3] Lynch M, Marinov GK (2015). The bioenergetic costs of a gene. Proc Natl Acad Sci U S A.

[4] Lynch M, Marinov GK (2017). Membranes, energetics, and evolution across the prokaryote-eukaryote divide. eLife.

[5] Lynch M, Marinov GK (2016). Mitochondria do not boost the bioenergetic capacity of eukaryotic cells. Proc Natl Acad Sci U S A.

[6] Lane N, Martin WF (2016). Mitochondria, complexity and evolutionary deficit spending. Proc Natl Acad Sci U S A.

[7] Martin WF, Müller M (1998). The hydrogen hypothesis for the first eukaryote. Nature.

[8] Lane N, Martin WF (2010). The energetics of genome complexity. Nature.

[9] Müller M (2012). Biochemistry and evolution of anaerobic energy metabolism in eukaryotes. Microbiol Mol Biol Rev.

[10] Gould SB, Garg SG, Martin WF (2016). Bacterial vesicle secretion and the evolutionary origin of the eukaryotic endomembrane system. Trends Microbiol.

[11] Martin WF (2017). Symbiogenesis, gradualism and mitochondrial energy in eukaryote evolution. Period Biol.

[12] Martin WF, Tielens AGM, Mentel M, Garg SG, Gould SB (2017). The physiology of phagocytosis in the context of mitochondrial origin. Microbiol Mol Biol Rev.

[13] Helmholtz H (1847). Über die Erhaltung der Kraft, eine physikalische Abhandlung.

[14] Rubner M (1902). Die Gesetze des Energieverbrauchs bei der Ernäherung.

[15] Battley EH (1987). Energetics of Microbial Growth.

[16] Stouthamer AH (1973). A theoretical study on the amount of ATP required for synthesis of microbial cell material. Antonie Van Leeuwenhoek.

[17] Feist AM (2007). A genome-scale metabolic reconstruction for Escherichia coli K-12 MG1655 that accounts for 1260 ORFs and thermodynamic information. Mol Syst Biol.

[18] Akashi H, Gojobori T (2002). Metabolic efficiency and amino acid composition in the proteomes of Escherichia coli and Bacillus subtilis. Proc Natl Acad Sci U S A.

[19] Craig CL, Weber RS (1998). Selection costs of amino acid substitutions in ColE1 and ColIa gene clusters harbored by Escherichia coli. Mol Biol Evol.

[20] Lenski RE (2023). Revisiting the design of the long-term evolution experiment with Escherichia coli. J Mol Evol.

[21] Lynch M (2024). The bioenergetic cost of building a metazoan. Proc Natl Acad Sci U S A.

[22] Lynch M (2007). The frailty of adaptive hypotheses for the origins of organismal complexity. Proc Natl Acad Sci U S A.

[23] Gerlitz M (2018). Elusive data underlying debate at the prokaryote eukaryote divide. Biol Direct.

[24] Neidhardt FC, Ingraham JL, Schaechter M (1990). Physiology of the bacterial cell.

[25] Scott M (2010). Interdependence of cell growth and gene expression: origins and consequences. Science.

[26] Laskowski RA, Gerick F, Thornton JM (2009). The structural basis of allosteric regulation in proteins. FEBS Lett.

[27] Allen JF (2015). Why chloroplasts and mitochondria retain their own genomes and genetic systems: colocation for redox regulation of gene expression. Proc Natl Acad Sci U S A.

[28] Schavemaker PE, Munoz-Gomez SA (2022). The role of mitochondrial energetics in the origin and diversification of eukaryotes. Nat Ecol Evol.

[29] Munoz-Gomez SA (2023). Energetics and evolution of anaerobic microbial eukaryotes. Nat Microbiol.

[30] Muñoz-Gómez SA (2024). The energetic costs of cellular complexity evolution. Trends Microbiol.

[31] Phillips R, Milo R (2009). A feeling for the numbers in biology. Proc Natl Acad Sci U S A.

[32] Phillips R (2013). Physical Biology of the Cell.

[33] Thauer RK, Jungermann K, Decker K (1977). Energy conservation in chemotrophic anaerobic bacteria. Bacteriol Rev.

[34] Berg JM, Stryer L, Tymoczko JL, Gatto GJ (2015). Biochemistry.

[35] Farmer IS, Jones CW (1976). The energetics of Escherichia coli during aerobic growth in continuous culture. Eur J Biochem.

[36] Stouthamer AH, Bettenhausen CW (1977). A continuous culture study of an ATPase-negative mutant of Escherichia coli. Arch Microbiol.

[37] Kayser A (2005). Metabolic flux analysis of Escherichia coli in glucose-limited continuous culture. I. Growth-rate dependent metabolic efficiency at steady state. Microbiology.

[38] Maitra A, Dill KA (2015). Bacterial growth laws reflect the evolutionary importance of energy efficiency. Proc Natl Acad Sci U S A.

[39] Wilson EB (1928). The Cell in Development and Heredity.

[40] Wallin IE (1925). On the nature of mitochondria. IX. Demonstration of the bacterial nature of mitochondria. Am J Anat.

[41] Mereschkowsky C (1905). Über Natur und Ursprung der Chromatophoren im Pflanzenreiche. Biol Centralblatt.

[42] Sagan L (1967). On the origin of mitosing cells. J Theor Biol.

[43] Raff RA, Mahler HR (1972). The non symbiotic origin of mitochondria. Science.

[44] Cavalier-Smith T (1975). The origin of nuclei and of eukaryotic cells. Nature.

[45] Bogorad L (1975). Evolution of organelles and eukaryotic genomes. Science.

[46] Uzzel T, Spolsky C (1974). Mitochondria and plastids as endosymbionts: a revival of special creation?. Am Sci.

[47] Crockford PW (2023). The geologic history of primary productivity. Curr Biol.

[48] Martin WF, Hoffmeister M, Rotte C, Henze K (2001). An overview of endosymbiotic models for the origins of eukaryotes, their ATP-producing organelles (mitochondria and hydrogenosomes), and their heterotrophic lifestyle. Biol Chem.

[49] Martin WF, Garg S, Zimorski V (2015). Endosymbiotic theories for eukaryote origin. Phil Trans Roy Soc Lond B.

